# Engineering the Colloidal Properties of Iron Oxide Nanoparticles for High *T*_1_ MRI Contrast at 64 mT

**DOI:** 10.1021/acsanm.5c03154

**Published:** 2025-09-12

**Authors:** Samuel D. Oberdick, Gabriella G. Erich, Arabella R. Stockdale, Kaitlyn M. Betz, Kalina V. Jordanova, Andrew G. Korovich, O. Thompson Mefford, Giacomo Parigi, Megan E. Poorman, Gary Zabow, Kathryn E. Keenan

**Affiliations:** † Department of Physics, University of Colorado, Boulder, Colorado 80309, United States; ‡ 10833 National Institute of Standards and Technology, Boulder, Colorado 80305, United States; § Department of Materials Science and Engineering, 2545 Clemson University, Clemson, South Carolina 29634, United States; ∥ Radiological Sciences Laboratory, Stanford University, Stanford, California 94305, United States; ⊥ National Institute of Standards and Technology, Gaithersburg, Maryland 20899, United States; # Magnetic Resonance Center (CERM), University of Florence, via Luigi Sacconi 6, Sesto Fiorentino, Florence 50019, Italy; ¶ Department of Chemistry “Ugo Schiff”, University of Florence, via della Lastruccia 3, Sesto Fiorentino, Florence 50019, Italy; ∇ Consorzio Interuniversitario Risonanze Magnetiche Metallo Proteine (CIRMMP), via Luigi Sacconi 6, Sesto Fiorentino, Florence 50019, Italy; ○ Hyperfine, Inc. Guilford, Guilford, Connecticut 06437, United States

**Keywords:** magnetic nanoparticles, contrast agents, medical imaging, magnetic resonance imaging, iron oxide nanoparticles, MRI contrast

## Abstract

Low-field magnetic resonance imaging (LF-MRI), an emerging form of portable and accessible MRI, has tremendous potential for point-of-care diagnostics and democratization of medical imaging. As LF-MRI evolves, there is a need to develop workflows, materials, and technologies designed specifically for the low-field regime. Here, monodisperse iron oxide nanoparticles (IONs) are evaluated for applications as positive contrast *T*_1_ agents using 64 mT LF-MRI. The nanoparticles were synthesized with relatively large diameters (16 and 22 nm) to promote high relaxivity at 64 mT. The particles were also stabilized with poly­(ethylene glycol) (PEG), a biocompatible ligand. The 16 nm particles showed an especially high longitudinal relaxivity, 90 L mmol^–1^ s^–1^, representing more than a 10× increase compared to a common Gd-based agent. The effects of colloidal stability were investigated by functionalizing the 22 nm particles with PEG ligands of varying molecular weights. The IONs displayed aggregation that depended on the length of the PEG ligands. The clustering reduced the longitudinal relaxivity of the ION-filled solutions.

## Graphical abstract

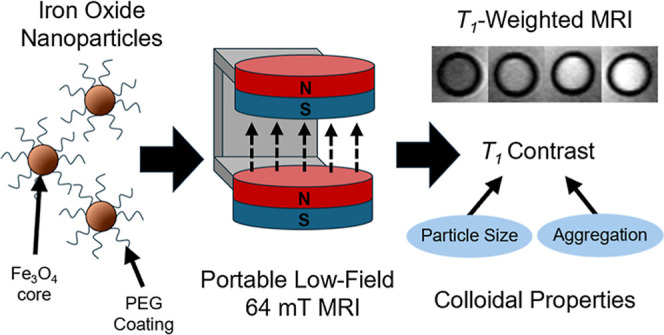

## Introduction

Low-field MRI (LF-MRI) technology is in the midst of a revolution.^1–4^ In 2020, a portable 64 mT LF-MRI scanner (Hyperfine Swoop, Guilford, CT, USA) was approved for neuroimaging by the United States (U.S.) Food and Drug Administration (FDA) and introduced to the U.S. commercial market. Meanwhile, there has been increasing interest in development and construction of LF-MRI systems.^5–11^ These new LF-MRI scanners are creating exciting opportunities for bedside point-of-care neuroimaging.^12–16^ The scanners may also contribute to the democratization of healthcare, since they can be used in regions lacking the resources and infrastructure for standard MRI scanners.^17,18^

MRI contrast agents are magnetic materials that can be used to selectively change the contrast of tissues, fluids, and organs. In standard clinical field strength MRI examinations (typically 1.5 to 3 T), contrast agents are used for about 25% of all procedures.^19^ As LF-MRI evolves, there may be a demand for contrast agents engineered specifically for use at lower field strengths. Iron oxide nanoparticles (IONs) have generated considerable interest as LF-MRI contrast agents.^2,20–25^

At 64 mT, IONs have a combination of structural and magnetic properties that make them promising materials for high relaxivity *T*_1_ contrast agents.^21^ *T*_1_ contrast agents are useful for MRI applications because they shorten *T*_1_ times and create bright regions on images. Regions containing *T*_1_ agents appear brighter than surrounding areas, which can enhance tissue differentiation. The shortened *T*_1_ times can also reduce scan times, allowing for more scan repetitions and increased signal-to-noise (SNR). While initial studies have been encouraging, additional research is needed to understand the relationships between magnetic, structural, and colloidal properties and resultant *T*_1_ contrast with 64 mT LF-MRI.

Additionally, the development of magnetic nanoparticle *T*_1_ agents has been driven by safety concerns about Gd-based agents, the primary contrast *T*_1_ agents used at 1.5 and 3 T. Evidence suggests that Gd-based agents can be potentially toxic and accumulate in the brain.^26,27^ Ultrasmall IONs (with diameters less than 5 nm) can be used as *T*_1_ agents at clinical fields strengths.^28–30^ However, specialized synthesis techniques are required to make particles with such small diameters. Manganese oxide (MnO) particles can also be used as *T*_1_ agents at clinical field strengths,^31,32^ but the *r*_1_ values can be low compared to Gd-based agents. The *r*_1_ values can be enhanced by embedding the MnO particles within polymer composite nanoparticles,^33^ but the synthesis of these composite particles requires multiple synthesis steps. Until recently, research on magnetic nanoparticle *T*_1_ contrast agents has focused largely on clinical fields strengths. With the advent of LF-MRI, though, new research needs to be done to understand the structure–property relationships for magnetic nanoparticle contrast at lower fields.

Here, the properties of 16 and 22 nm IONs were characterized and evaluated for *T*_1_ contrast at 64 mT. Previous studies showed that, for IONs ranging in size from 5 to 16 nm, *r*_1_ increased as a function of size.^21^ Larger particles were measured in this study to better understand the relationship between particle size, structure, and 64 mT LF-MRI *T*_1_ contrast. As iron oxide particles get larger, they (1) have slower Néel relaxation rates (internal relaxation of magnetic moment) and (2) may aggregate more easily because of the particles’ larger magnetic moments. This manuscript explores the effects of reduced Néel relaxation rates and aggregation on 64 mT LF-MRI *T*_1_ contrast. The particles were coated with poly­(ethylene glycol) (PEG), a biocompatible and water-soluble ligand. The 16 nm particles had an especially high longitudinal relaxivity, displaying a greater than 10-fold increase compared to a common Gd-based *T*_1_ agent. Nuclear magnetic relaxation dispersion (NMRD) measurements showed that the reduced Néel relaxation rate (corresponding to an increase in relaxation time) in 22 nm IONs influenced the measured relaxivity compared to the 16 nm IONs. The effect of aggregation and colloidal stability was studied by coating the 22 nm particles with PEG ligands of varying molecular weights. Depending on the PEG length, the IONs showed different degrees of clustering, which reduced the relaxivity at 64 mT.

In conjunction with earlier research,^21^ these results can help guide the future design and colloidal engineering of ION-based contrast agents for LF-MRI.

## Experimental Section

### Materials

Iron­(III) acetylacetonate, 3,4-dihydroxy-dl-phenylalanine (dl-DOPA; crystalline, 98%), and sodium nitrite (≥97%) were purchased from Alfa Aesar. Linear monofunctional poly­(ethylene glycol) methyl ether with average *M*_n_ weights of 2000 Da, 5000 Da, 10,000 and 20,000 Da were purchased from JenKEM (*M*_n_ refers to the number-average molecular weight). Linear monofunctional *N*-hydroxysuccinimide (NHS) terminated PEG of molecular weight 10,000 Da was purchased from Creative PEGworks. 4-(Dimethylamino)­pyridine (≥99%), NHS (98%), *N*,*N*′-dicyclohexycarbodiimide, succinic anhydride (≥99%), diethyl ether (>95%), chloroform (99.8%), agarose, poly­(ethylene glycol) diacrylate (average *M*_n_ 700), 2,2-dimethoxy-2-phenylacetophenone, and oleic acid were purchased from Sigma-Aldrich. Methanol, ethanol (anhydrous, histological grade), tetrahydrofuran (99%, extra dry over molecular sieves), and hexanes (99.3%) were purchased from Fisher Chemical. Dimethylformamide (99.8%, extra dry over molecular sieves) and azobis­(isobutyronitrile) were purchased from Acros Organics.

### Magnetic Nanoparticle Synthesis

Magnetite particles of 16 and 22 nm diameters were prepared using a modified one pot thermal decomposition method first presented by Sun and Zeng.^34^ Magnetite particles were produced by adding iron­(III) acetylacetonate (1.06 g, 0.003 mmol) and oleic acid (15 mL, 0.0475 mmol) to a three neck round-bottom flask under a nitrogen blanket with a flow rate 0.2 L/min. The three neck flask was placed into a low melting point solder bath, which had been preheated to 250 °C. The solution was heated at a ramp rate of 10 °C/min from 250 to 350 °C in the solder bath and held isothermally for 2 h. The solution was annealed in open air at 250 °C for 1.5 h to oxidize the nanoparticles into the magnetite phase. An air condenser was employed in the isothermal step for the 16 nm particle synthesis. The condenser was removed during the annealing process. No condenser was used for the 22 nm synthesis. The resulting particles were purified by precipitating the particles in a 1:3:6 ratio of particles in hexane, ethanol, and acetone. Centrifugation isolated the product on the walls which was then redispersed in hexane. This process was repeated four times.

### Ligand Exchange of Magnetite Nanoparticles

To create aqueous particle dispersions, the hydrophobic ligand coating on the surface of the particle was exchanged with PEG-nitro-3,4 dihydroxyphenylanaline (nitroDOPA). The syntheses of nitroDOPA and PEG-nitroDOPA are described in the Supporting Information (Sections S1 and S2). This process was performed for each molecular weight sample using the same batch of magnetite nanoparticles. To remove any excess oleic acid, particles suspended in hexane were washed with ethanol, acetone, and diethyl ether three times before removing the solvent through rotary evaporation. The cleaned particles (45 mg) were dried and redispersed in dichloromethane (DCM) (5 mL) with sonication until a stable colloid was formed. PEG-nitroDOPA (0.04 mmol) was also dissolved in 5 mL DCM and sonicated. The nanoparticle suspension was then added dropwise (5 mL, at 9 mg/mL) to the PEG solution while sonicating for 30 min. The resulting mixture was agitated on a shake plate for 48 h. The coated particles were precipitated from solution using a 1:8 volume ratio of DCM and cold diethyl ether. This solution was centrifuged, magnetically decanted, and redispersed until the runoff ran clear. This process is critical for eliminating any agglomerated particles or leftover oleic acid. Collected particles were dispersed in deionized (DI) water and dispersed with sonication. Dialysis against water was performed for 36 h with frequent water changes to remove excess polymer in the solution.

### Magnetometry

Magnetometry was performed using a Quantum Design MPMS3 magnetometer. The magnetic moment as a function of applied field was collected at 300 K for each of the samples. The magnetic moment was converted to magnetization by assuming the particles were made of pure magnetite and using the measured iron concentration. Samples were prepared by mixing 10 μL of ION solution with 90 μL of ultraviolet light-curable photopolymer. The photopolymer was poly­(ethylene glycol) diacrylate (average *M*_n_ 700) mixed with a photoinitiator. The photoinitiator, 2,2-dimethoxy-2-phenylacetophenone, was added to the poly­(ethylene glycol) diacrylate in a mass fraction of 0.01. The samples were photocured under N_2_ gas for 3 min.

### MRI Measurements

MRI measurements were performed with a 64 mT scanner (Hyperfine Swoop). Samples were prepared for MRI measurements by diluting stock solutions of IONs in agarose gel (0.01 mass fraction agarose in water). Samples were prepared in 50 mL centrifuge tubes with 5 concentrations, ranging from approximately 0.01 mmol Fe/L to 0.05 mmol Fe/L, and loaded into a sample holder for MRI measurements. Concentration measurements were performed later to find the exact concentration of iron (mmol Fe) in the samples (see “Concentration Measurements”). *T*_1_ measurements were made using a research version of the Hyperfine proprietary *T*_1_-weighted inversion recovery 3D fast spin echo (FSE) sequence with a 220 mm × 180 mm × 180 mm field of view, an in-plane resolution of 1.6 mm × 1.6 mm, and a slice thickness of 5 mm. The inversion times used for the sequence were 100, 200, 300, 400, 500, 600, 700, 800, 900, 1100, 1300, 1500, 1800, 2100, and 2500 ms. The repetition time was 3000 ms, and the echo time was 7 ms. *T*_2_ measurements were performed using a research version of the Hyperfine proprietary *T*_2_ mapping sequence with a 220 mm × 180 mm × 180 mm field of view, an in-plane resolution of 1.5 mm × 1.5 mm, and a slice thickness of 5 mm. Echo times used for *T*_2_ acquisition were 7.29, 51.03, 94.77, 138.51, 182.25, 225.99, 269.73, 313.47, 357.21, 400.95, 444.69, 488.43, 532.17, 575.91, 619.65, 663.39, 707.13, 750.87, 794.61, and 838.35 ms. The repetition time was 3000 ms. All measurements were performed at an ambient lab temperature of 21.5 °C.

### *T*_1_/*T*_2_ Mapping and MRI Analysis

The MRI data was analyzed using a previously described custom Python workflow.^21^

### Dynamic Light Scattering

Dynamic light scattering (DLS) measurements were performed using a Malvern Zetasizer Nano. The standard deviation, σ, was calculated from the measured polydispersity index (PdI) using $PdI=\sigma^{2}/{\left(z‐average\;D_{h}\right)}^{2}$ , where “*z*-average *D*_*h*_” is the measured *z*-average hydrodynamic diameter.

### Transmission Electron Microscopy (TEM)

TEM was performed using a Hitachi HT7830 UHR 120 kV microscope. TEM samples were prepared using carbon-coated TEM grids. Particle size distributions (Section S3, Figure S3a,b) were acquired from microscope images using ImageJ.^35^

### Concentration Measurements

The iron concentration of ION samples was determined using inductively coupled optical emission spectroscopy (ICP-OES). The measurements were performed using a Perkin Elmer Optima 8300 ICP-OES optical system with a segmented-array charge-coupled device detector.

### Nuclear Magnetic Relaxation Dispersion (NMRD) Measurements

NMRD measurements were performed with a Stelar Spinmaster fast field cycling (FFC) relaxometer operating at approximately 20 °C. The prepolarization and acquisition fields were 15 MHz (0.35 T), with a prepolarization time of 0.2 s in the prepolarized experiments. The recycle delay was 0.2 s. Experiments were run as echo-detected experiments, with an echo delay of 40 μs. Triplicate measurements were collected from Larmor frequencies of 0.01 to 20 MHz, with a crossover to the prepolarized relaxation recovery sequence at 4 MHz from the inversion–recovery type experiment used at higher fields.

## Results and Discussion

### Structural and Magnetic Properties of the IONs

For this study, nanoparticles with relatively large core diameters (16 and 22 nm) were synthesized and functionalized with PEG, a biocompatible ligand. The nanoparticle sizes were chosen because previous research showed that 16 nm IONs exhibited high longitudinal relaxivities with 64 mT MRI.^21^ There were several reasons for functionalizing the nanoparticles with PEG. PEG is often used to coat nanomaterials for biomedical applications yielding biocompatible, aqueously dispersible composite systems. While the measurements reported here were all performed in vitro, the measurements on a PEG-coated ION system may be useful for future in vivo applications. The PEG coating also provided a mechanism for tuning the colloidal stability of the system. The 22 nm particles were functionalized with PEG ligands of differing molecular weights (2 kDa, 5 kDa, 10 kDa, 20 kDa), which affected the aggregation state of the colloidal system. Finally, the PEG-coated nanoparticles were water-soluble and could therefore be dispersed in aqueous gels for MRI experiments.

Figure 1a,b show TEM images of the 16 nm ± 1 and 22 nm ± 3 nm particles (reported here as the mean of the particle diameter ± standard deviation). The images show the particles immediately after initial synthesis and coated with oleic acid (before they were functionalized with PEG). The nanoparticles were synthesized using a one-pot thermal decomposition method, which produces magnetite (Fe_3_O_4_) nanoparticles.^34^ The magnetite phase was confirmed with X-ray diffraction (Section S4, Figures S4 and S5). For MRI measurements, the IONs were coated with PEG, a water-soluble ligand, and transferred to water (Figure 2). Figure 1c shows a schematic of a single PEG-coated nanoparticle. Additional details of the synthesis and ligand exchange are contained in the Supporting Information (Sections S1 and S2).

**Figure 1:**
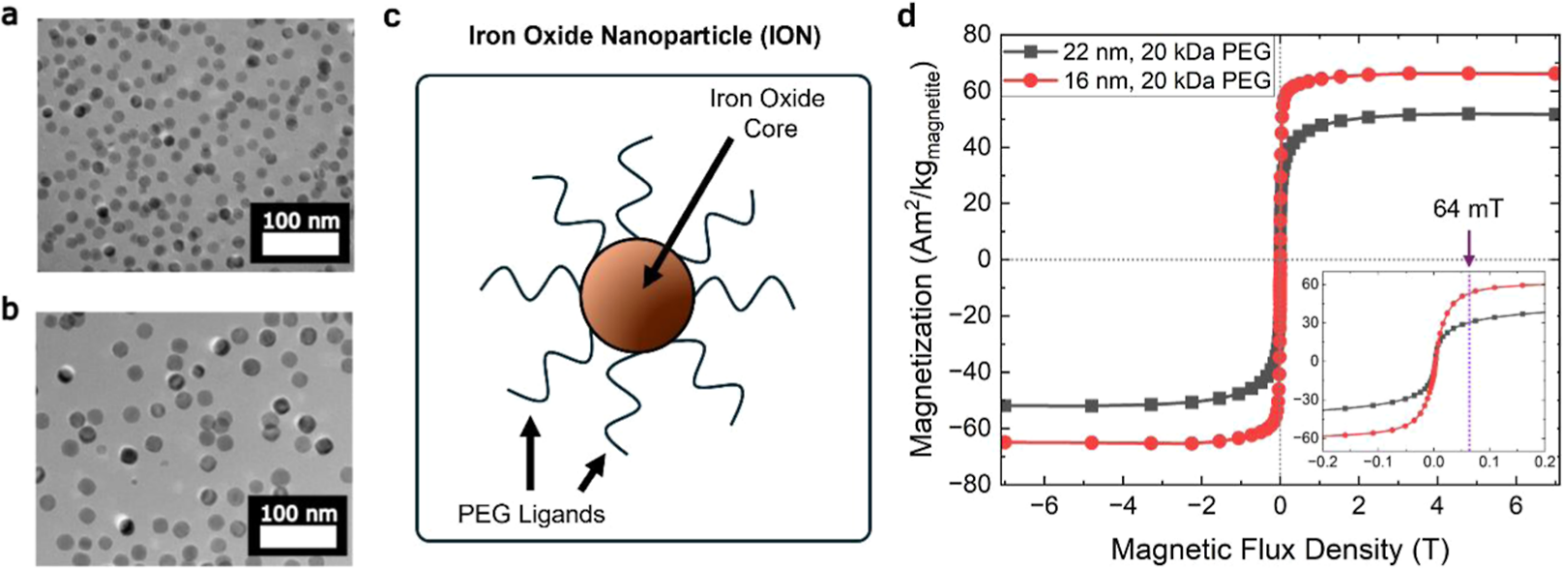
(a) Transmission electron microscope (TEM) image of 16 nm IONs. (b) TEM image of 22 nm IONs. (c) Schematic showing the structure of IONs used in this study. (d) Magnetization as a function of magnetic flux density from −7 T to +7 T (taken at 300 K). The inset shows a magnified region of magnetic flux density, from −0.2 T to +0.2 T, with an arrow indicating the 64 mT *B*_0_ of the LF-MRI scanner.

**Figure 2:**
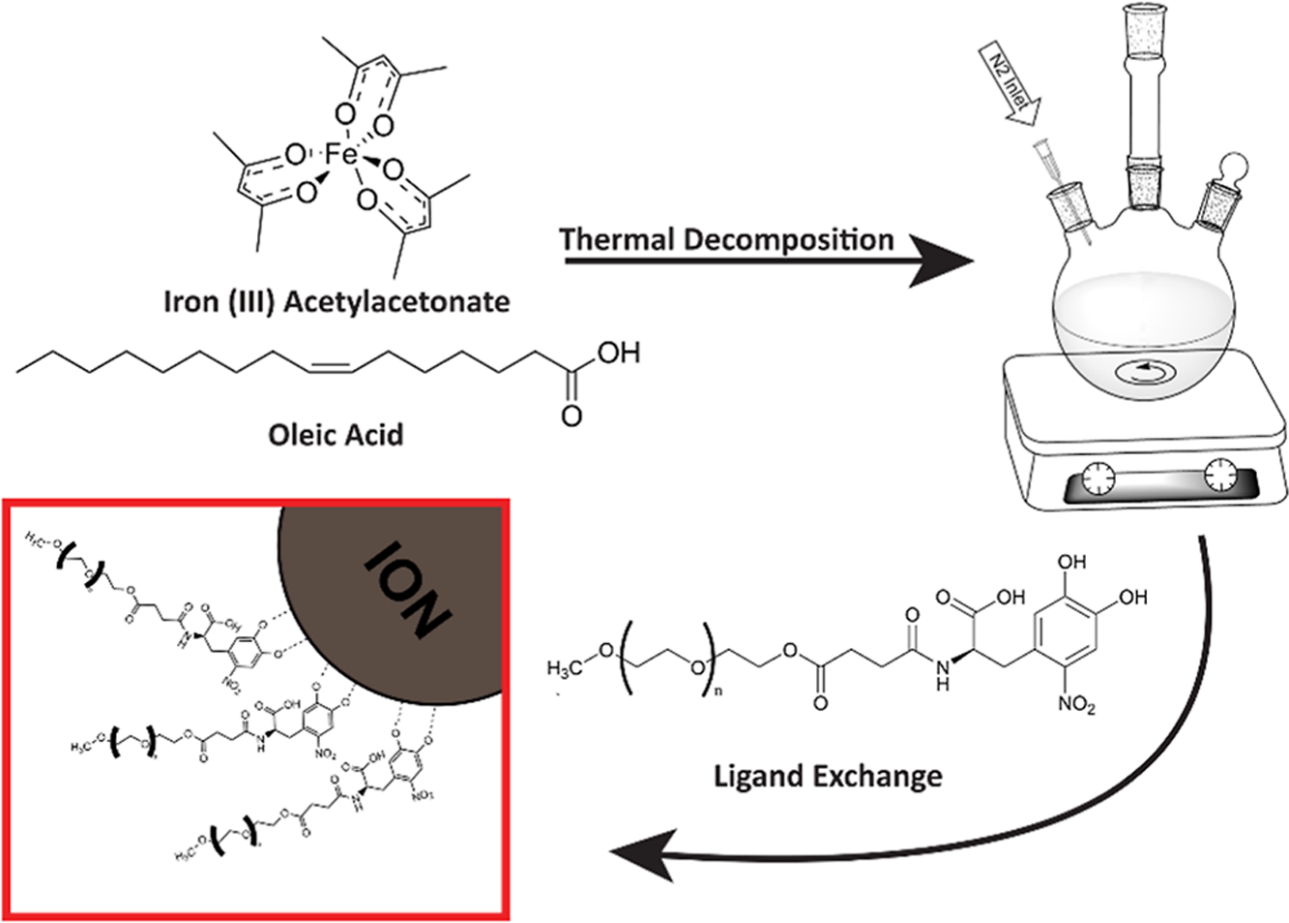
Synthesis of IONs. First, IONs were synthesized using thermal decomposition, producing nanoparticles coated with oleic acid. Then, a ligand exchange technique was used to coat the IONs with PEG. The PEG-coated nanoparticles were soluble in water.

Figure 1d shows magnetometry data (taken at 300 K) for two ION samples, the 22 nm IONs coated with 20 kDa PEG and the 16 nm IONs coated with 20 kDa PEG. The saturation magnetization of the samples (measured at 7 T) was 52 Am^2^/kg (roughly 57% of bulk magnetite) for the 22 nm IONs and 66 Am^2^/kg (roughly 72% of bulk magnetite) for the 16 nm IONs. The inset shows a magnified region from −200 mT to +200 mT, with a label for the +64 mT *B*_0_ strength of the LF-MRI scanner used in this study. At 64 mT, both ION samples reached more than 50% of their saturation magnetization.

### 64 mT MRI

The IONs were embedded in agarose gel (0.01 mass fraction of agarose in water) for 64 mT MRI measurements. The agarose served two functions. First, it prevented unwanted sedimentation and aggregation of the nanoparticles. Second, the agarose acted as a water-filled tissue mimicking environment.

The longitudinal and transverse relaxivities (*r*_1_ and *r*_2_) were measured for each ION sample using 64 mT MRI. First, samples were prepared with a systematic range of iron concentrations (and, correspondingly, ION concentrations). The measured iron concentrations had standard deviations ranging from 0.5% to 3.8% of the mean. Then, the samples were scanned with MRI and the data was used to measure the longitudinal and transverse relaxation times, *T*_1_ and *T*_2_. The measured relaxation times (*T*_1_ and *T*_2_) had standard deviations ranging from 0.4% to 6.8% of the mean. Next, the longitudinal and transverse proton relaxation rates, *R*_1_ = 1/*T*_1_ and *R*_2_ = 1/*T*_2_, were calculated. Finally, the data was fit using the following formulas

1 $$\frac{1}{T_{1}}=r_{1}\times \left[Fe\right]+\frac{1}{T_{1,agarose}}$$

2 $$\frac{1}{T_{2}}=r_{2}\times \left[Fe\right]+\frac{1}{T_{2,agarose}}$$

In eqs 1 and 2, [Fe] is the iron concentration of the sample (in mmol of iron atoms), *T*_1,agarose_ is the longitudinal relaxation time of the pure agarose sample (no IONs), and *T*_2,agarose_ is the transverse relaxation time of the pure agarose sample (no IONs). To find *r*_1_ and *r*_2_, the data for the relaxation rate (1/*T*_1_ or 1/*T*_2_) as a function of [Fe] was fit by a line, and the slope was reported as *r*_1_ and *r*_2_. Figure 3 shows an example of the *r*_1_ measurement process for the 16 nm IONs (coated with 20 kDa PEG). Additional details on the fits are provided in the Supporting Information (Section S5, Figures S6 and S7).

**Figure 3:**
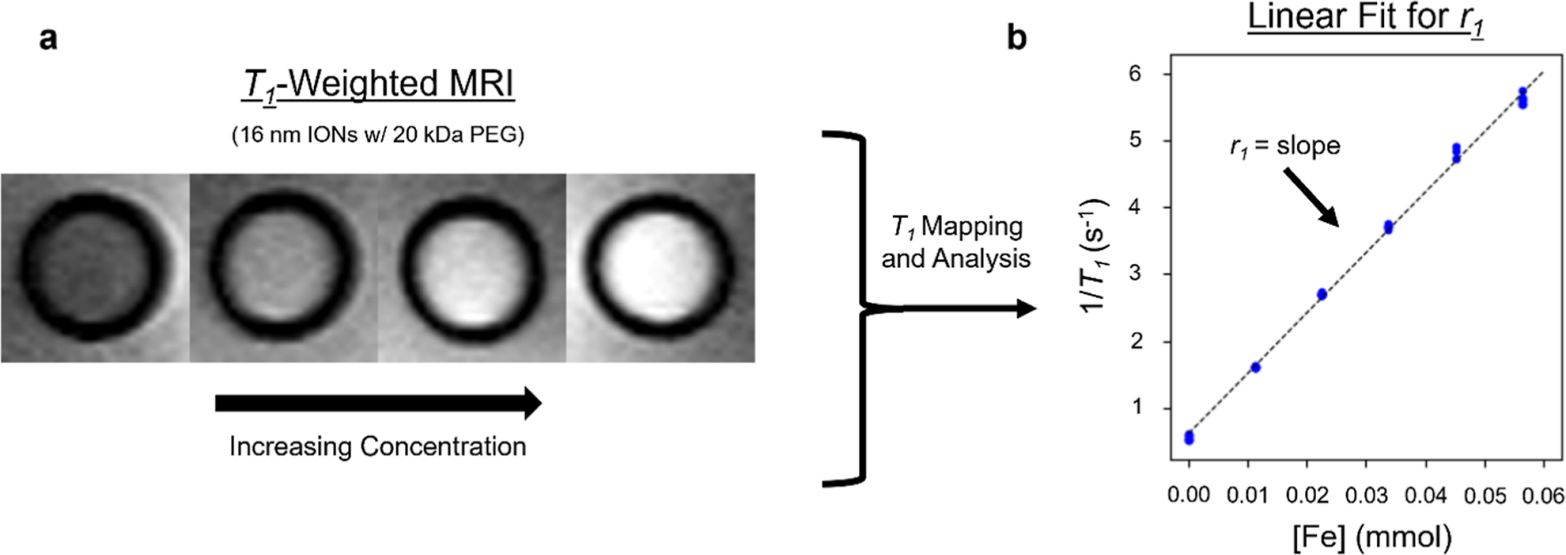
Process for measuring the longitudinal relaxivity, *r*_1_. (a) *T*_1_-weighted MRI of 16 nm IONs (coated with 20 kDa PEG) at increasing concentrations (inversion recovery 3D fast spin echo sequence with a 220 mm × 180 mm × 180 mm field of view, an in-plane resolution of 1.6 mm × 1.6 mm, a slice thickness of 5 mm, an inversion time of 400 ms, a repetition time of 3000 ms, and an echo time of 7 ms). (b) The MRI data was analyzed to find the longitudinal relaxation time, *T*_1_, and corresponding relaxation rate, 1/*T*_1_. The relaxation rate was plotted as a function of concentration and fit with a line. The slope of the line gave the longitudinal relaxivity.

The relaxivity is a useful gauge of the effectiveness of a contrast agent—it indicates how much the relaxation rate changes per change in the concentration of the material. If the relaxivity is a large value, it means that the material efficiently induces proton relaxation (in terms of the quantity of material used). For the ION samples reported on here, a large *r*_1_ indicates good potential as a *T*_1_ contrast agent.

Figure 4 shows *r*_1_, *r*_2_, and the ratio of *r*_2_/*r*_1_ for two different sizes of IONs (each coated with 20 kDa PEG). Figure 4 also includes the relaxivities of gadobenate dimeglumine (Gd-BOPTA), for comparison to a commonly used clinical Gd contrast agent (commercial name Multihance). The Gd-BOPTA was measured using 64 mT MRI in a previous study.^21^ The IONs showed a notable increase in *r*_1_ compared to Gd-BOPTA at 64 mT. The 16 nm nanoparticles had the highest *r*_1_, 90 L mmol^–1^ s^–1^, which was more than 10 times larger than Gd-BOPTA. The IONs also had significantly larger *r*_2_ values at 64 mT, compared to Gd-BOPTA. Despite the large *r*_2_ values, the ratio of the transverse to longitudinal relaxivities, *r*_2_/*r*_1_, was 1.4.

**Figure 4:**
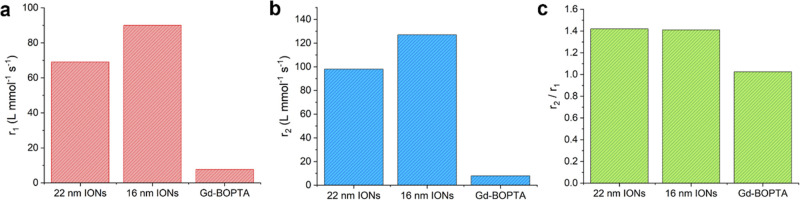
Magnetic resonance imaging (MRI) data collected with 64 mT scanner at ambient laboratory temperature. Data is presented for two particle sizes, 16 and 22 nm. Both sizes were coated with 20 kDa PEG. (a) Longitudinal relaxivity, *r*_1_, at 64 mT for 22 nm IONs, 16 nm IONs, and gadobenate dimeglumine Gd-BOPTA. (b) Transverse relaxivity, *r*_2_, at 64 mT for 22 nm IONs, 16 nm IONs, and Gd-BOPTA. (c) Ratio of transverse to longitudinal relaxivities, *r*_2_/*r*_1_, for 22 nm IONs, 16 nm IONs, and Gd-BOPTA. The values for Gd-BOPTA were measured in a previous study.^21^

The nanoparticles were also functionalized with varying molecular weights of PEG ligands and measured with 64 mT MRI. Figure 5 shows *r*_1_ and *r*_2_ data for nanoparticles with the same 22 nm core size but different sizes of PEG coatings. The PEG coatings ranged in molecular weight from 2 kDa to 20 kDa. Both *r*_1_ and *r*_2_ showed a dependence on the molecular weight of PEG. In both cases, the relaxivities decreased as the molecular weight was decreased.

**Figure 5:**
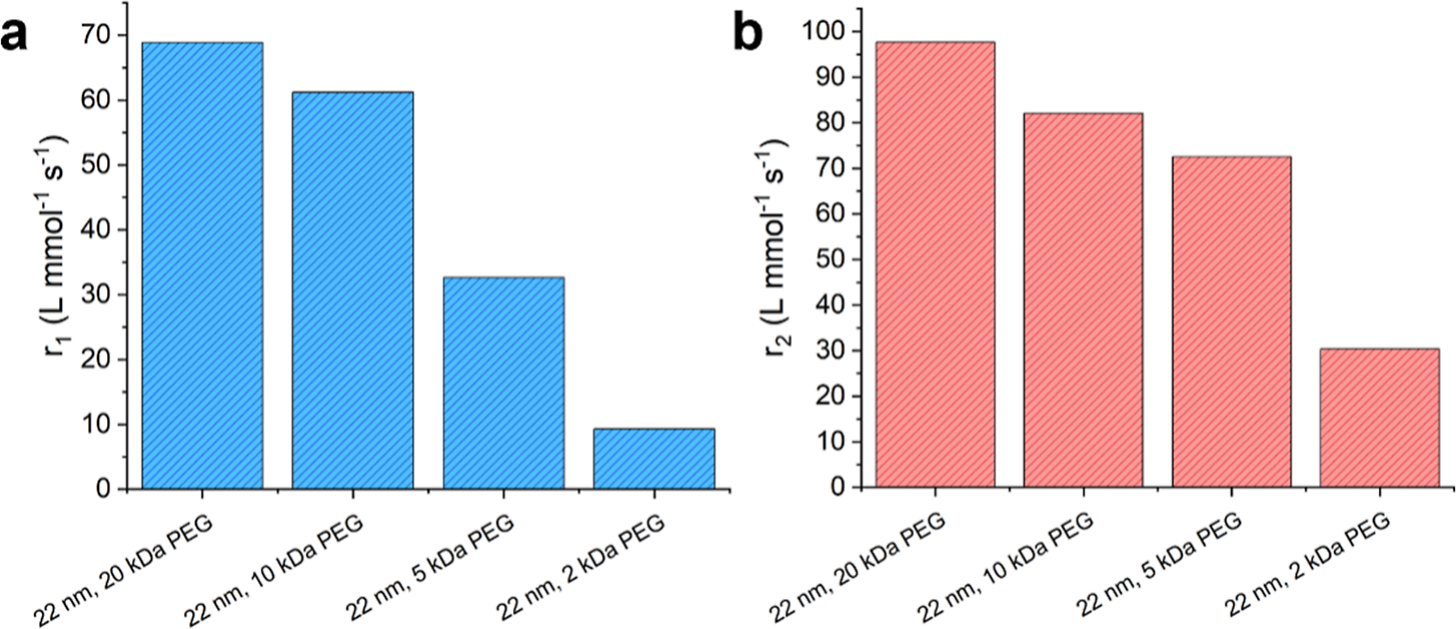
(a) Longitudinal relaxivity, *r*_1_, for 22 nm IONs functionalized with varying molecular weights of PEG. (b) Transverse relaxivity, *r*_2_, for 22 nm IONs functionalized with varying molecular weights of PEG.

Table 1 contains a summary of all 64 mT MRI data for the various samples.

**Table 1:** 64 mT MRI Longitudinal Relaxivity (*r*_*1*_), Transverse Relaxivity (*r*_*2*_), and the Ratio *r*_*2*_/*r*_*1*_ for all ION Samples

sample description	*r*_1_ (L mmol^–1^ s^–1^)	*r*_2_ (L mmol^–1^ s^–1^)	*r*_2_/*r*_1_
16 nm ION core, 20 kDa PEG coating	90	127	1.4
22 nm ION core, 20 kDa PEG coating	69	98	1.4
22 nm ION core, 10 kDa PEG coating	61	83	1.4
22 nm ION core, 5 kDa PEG coating	33	73	2.2
22 nm ION core, 2 kDa PEG coating	9	28	3.1

### Dynamic Light Scattering (DLS) and Evidence of Cluster Formation

DLS was used to characterize the hydrodynamic diameter of the 22 nm IONs in aqueous solution. Figure 6a shows the intensity-weighted distribution of hydrodynamic diameters for all 22 nm ION samples. Somewhat counterintuitively, the IONs with the lowest PEG molecular weights had the largest hydrodynamic diameters. A possible hypothesis is that the shorter PEG allowed the nanoparticles to form aggregates. In general, the colloidal stability of particulate suspensions depends on the length and density of stabilizing ligands, and smaller ligands can lead to reduced steric repulsion and lower colloidal stability. This hypothesis is also supported by the observation of large clusters (*D*_*h*_ greater than 1 μm). The ION samples with the greatest fraction of large clusters also had the smallest molecular weight of PEG.

**Figure 6:**
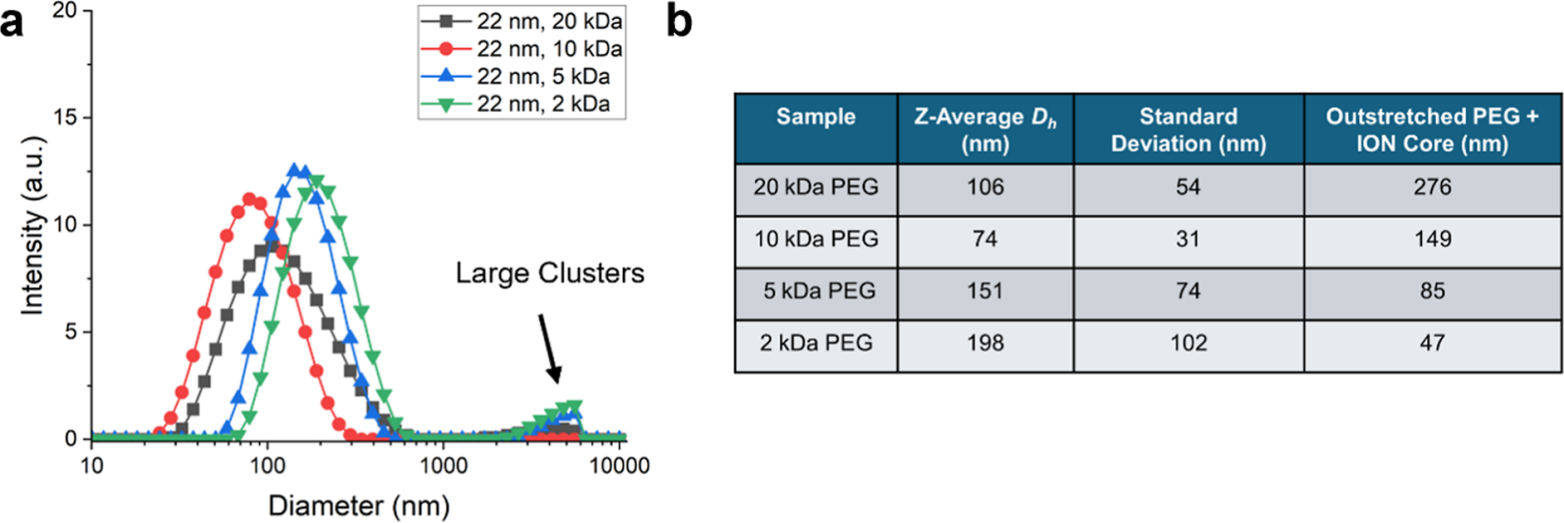
(a) DLS intensity-weighted size distributions of hydrodynamic diameters for 22 nm ION cores functionalized with PEG of varying molecular weights. (b) Table with z-average hydrodynamic diameter, standard deviation of z-average hydrodynamic diameter, and theoretical prediction for size of outstretched PEG and ION core.

Figure 6b shows a table of measured z-average values of the hydrodynamic diameter, *D*_*h*_, from the DLS. Figure 6b also contains predicted values for the PEG/ION diameter (surfactant and core), which were calculated for the extreme case where the PEG had been fully extended. Details of the calculation are contained in the Supporting Information (Section S6 and Table S1). For the larger molecular weights (10 kDa and 20 kDa), the measured *D*_*h*_ was smaller than the theoretical value of fully extended PEG attached to the 22 nm ION core. The difference is likely because PEG molecules will not naturally extend to their full length in solution. Instead, they will adopt some sort of compressed conformation. A comparison of the IONs with lower PEG molecular weight, however, showed a measured *D*_*h*_ that was greater than the size of fully extended PEG molecules attached to the 22 nm ION core. This suggests that the samples with lower PEG molecular weights have formed aggregates containing at least a few particles.

### Nuclear Magnetic Relaxation Dispersion (NMRD) Measurements

The longitudinal relaxivity, *r*_1_, was measured as a function of Larmor frequency from 0.01 to 20 MHz using NMRD. Figure 7a shows data for the 16 nm IONs functionalized with 20 kDa PEG. The data shows a broad peak centered around approximately 1 MHz. Figure 7b shows data for the 22 nm IONs functionalized with PEG ranging in molecular weight from 2 kDa to 20 kDa. The NMRD curves for the 22 nm IONs do not show a peak over the measured frequency range. Also, the amplitude of the spectrum depended on the molecular weight of the PEG. The amplitude decreased as the molecular weight of the PEG decreased. For all NMRD data, the *r*_1_ values measured at 2.7 MHz (64 mT) were slightly larger (∼1.1×) than those measured with the 64 mT MRI. The difference between the two measurements may be due to differences in measurement temperature, differences in the homogeneity of the samples, or errors in the iron concentration.

Figure 7a,b also show theoretical calculations of *r*_1_ as a function of proton Larmor frequency for 16 and 22 nm IONs. The theoretical curves were generated using the model developed by Roch et al.,^36^ which has been commonly used to describe NMRD measurements for ION-based colloidal systems.^37–41^ While the theory has been successfully applied to many ION-based systems, there are specific situations where modified theories may be needed. For instance, recent research suggests that, for IONs with certain types of electrostatic stabilization, an inner–sphere interaction can explain pH dependence of ION-induced MRI contrast.^42^ The parameters used to generate the curves were informed by structural and magnetic properties of the IONs (Section S7, Tables S2 and S3). The calculations for the 16 nm particles matched the data well at 64 mT but overestimated the relaxivity at lower frequencies. The calculations for the 22 nm particles overestimated *r*_1_ at low frequencies and underestimated r_1_ at higher frequencies. The mismatch between theory and experiment could have been caused by several factors. Aggregation of the particles may have had nonuniform effects on the NMRD curve (influencing certain frequency ranges more than others). Also, the IONs were not ideally monodispersed, and the distribution in particle sizes could have impacted the match between experiment and theory.

**Figure 7:**
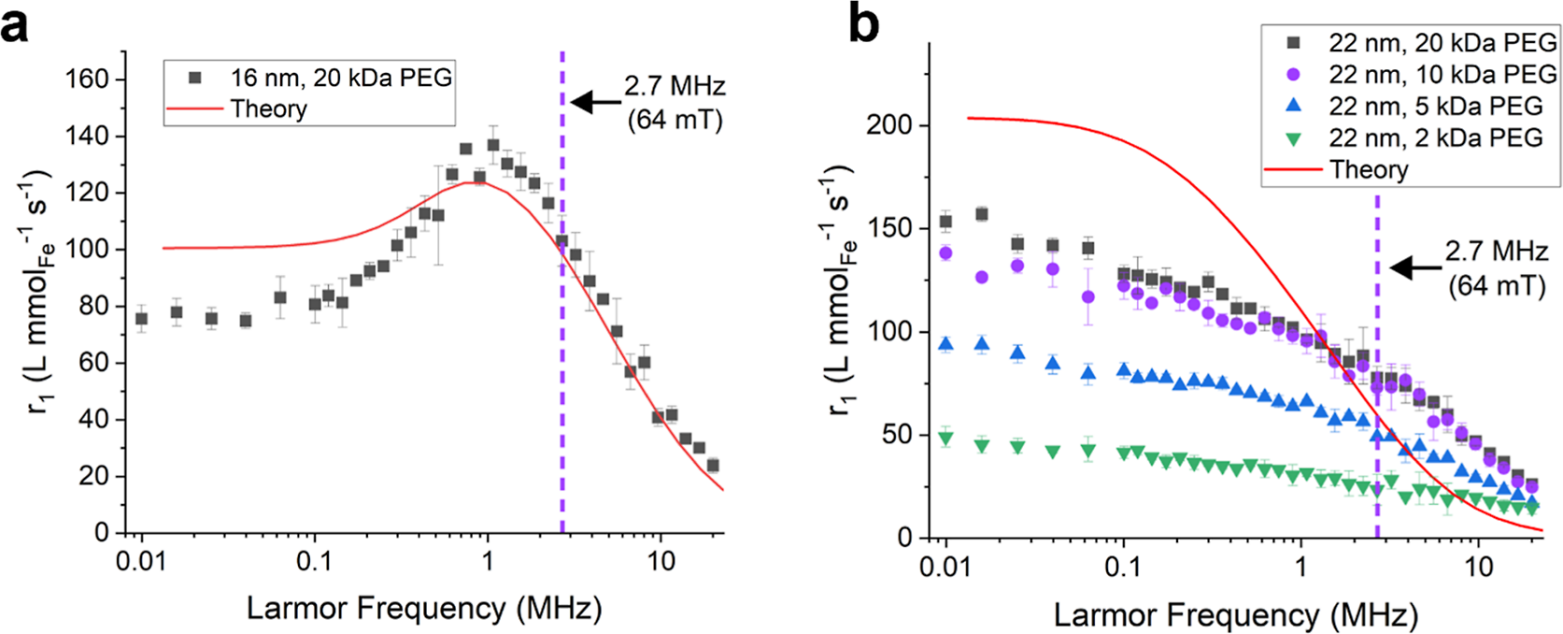
NMRD data for *r*_1_ from 0.01 to 20 MHz for (a) 16 nm IONs functionalized with 20 kDa PEG and (b) the 22 nm IONs functionalized with PEG ranging from 2 kDa to 20 kDa. Each sample was measured three times. The error bars represent the standard deviation of the three measurements. The solid lines show theoretical calculations for the longitudinal relaxivity, *r*_1_, as a function of proton Larmor frequency.

The difference in qualitative appearance of NMRD curves for the 16 nm IONs and 22 nm IONs was due to the difference in particle diameter and corresponding Néel relaxation time (the time scale for thermally driven fluctuations of the particle’s internal magnetic moment). The Néel relaxation time for the 16 nm IONs was estimated to be about 50 ns and the relaxation time for the 22 nm IONs was estimated to be about 3 μs, which was many orders of magnitude larger (Section S8 and Table S4).

## Discussion

ION-based contrast agents have the capacity to perform well as LF-MRI *T*_1_ contrast agents, but their longitudinal relaxivity depends on colloidal properties like particle size and aggregation state. The sizes of IONs used in this study (16 and 22 nm) showed high *r*_1_ values when measured with 64 mT MRI. Notably, the 16 nm IONs exhibited an *r*_1_ (90 L mmol^–1^ s^–1^) that was more than 10× higher than a commonly used Gd-based contrast agent. The 22 nm IONs also showed a large relaxivity (69 L mmol^–1^ s^–1^ for the particles coated with 20 kDa PEG), though not as large as the 16 nm particles. A high relaxivity is important for contrast agent applications because it means that smaller quantities of material could be used to achieve diagnostically relevant contrast enhancement.

IONs with core sizes like those used in this study match important length and time scales for efficient longitudinal relaxation rates at low field.^21,36^ The NMRD data and corresponding theoretical calculations (Figure 7) provide insights into longitudinal proton relaxation from IONs in the low-field MRI regime. The relaxation is mediated by two processes: (1) at lower NMRD frequencies, proton relaxation is dominated by Néel fluctuations, which are thermally driven fluctuations in the magnetic moment of the nanoparticles; (2) at higher NMRD frequencies, proton relaxation is dominated by diffusion-modulated dipole–dipole interactions between the nuclear spin of the protons (which are attached to diffusing water molecules) and the average magnetic moment of the IONs.^37,43^ The shape of the NMRD curve is governed by a competition of these two processes.

The 16 nm IONs showed a broad peak centered about 1 MHz (Figure 7a). Theoretical predictions from the model of Roch et al.^36^ validated the shape of the curve. At 64 mT, the *r*_1_ was approximately 100 L mmol^–1^ s^–1^, which was slightly higher than 64 mT MRI measurements. The peak in the low-field regime was wide, extending from approximately 0.5 to 3 MHz. This suggests that particles with similar sizes could be used as *T*_1_ agents for scanners operating not only at 64 mT, but also at neighboring fields.

The 22 nm IONs showed an increase in *r*_1_ at low fields and no peak in the NMRD spectrum. Larger iron oxide nanoparticles will have a higher anisotropy energy, and a longer time scale for Néel fluctuations. NMRD spectra for particles with relatively long Néel times have large *r*_1_ values at low frequencies, creating the low-frequency plateau seen in Figure 7b.^43^

For relatively large particles, small changes in the diameter can lead to significant changes in the Néel times, due to the exponential dependence in particle volume. For example, the Néel time of the 16 nm particles was estimated to be about 50 ns, while the Néel time of the 22 nm particles was estimated to be about 3 μs. The increased Néel time of the larger 22 nm particles contributed to the qualitatively different shape of the NMRD curves for the larger particles.

Colloidal stability and ligand size should also be considered when trying to increase *r*_1_. Comparison of the DLS data and *r*_1_ data reported here suggest that colloidal aggregation of the IONs can significantly affect *r*_1_. The measurements on the 22 nm IONs functionalized with PEG ligands of varying size show that increased clustering leads to decreased *r*_1_ and *r*_2_ at 64 mT. The NMRD measurements showed a similar trend for *r*_1_. These results are consistent with previous research by Roch et al., which showed that *r*_1_ was significantly reduced when IONs aggregated on length scales of several hundred nanometers.^44^ This suggests that IONs with a higher degree of colloidal stability (and lower degree of clustering) will make better contrast agents. It can be difficult, however, to maintain colloidal stability in biological environments. Still, coating the IONs with large ligands may help preserve high relaxivity, even in crowded bioenvironments. Indeed, the results here showed that the IONs coated with the largest PEG ligands displayed the highest relaxivity.

The dependence between *r*_1_ and ION aggregation might be used to develop *T*_1_-based sensors for LF-MRI. ION-based sensors with reversible aggregation state have been previously developed for *T*_2_ readout of biologically relevant variables using larger, clinical MRI field strengths.^45–49^ Comparison of the DLS data and 64 mT MRI *r*_1_ data suggests that *r*_1_ can change significantly depending on aggregation state—the 22 nm particles showed an 87% difference between the 20 kDa PEG coating and the 2 kDa PEG coating. IONs could, therefore, be used as high-sensitivity LF-MRI sensors, if the colloidal aggregation state can be engineered for reversible clustering in response to environmental conditions.

While the results from this study provide a framework for designing ION-based LF-MRI contrast agents, more research will be needed to understand the relationships between in vivo colloidal stability and resultant LF-MRI contrast. Previous studies and reviews can be used to guide in vitro and in vivo research on ION toxicity,^50,51^ uptake and clearance,^52^ and MRI applications.^53^

## Conclusions

IONs have high potential for use as *T*_1_ contrast agents with 64 mT MRI. To design effective contrast agents, though, the colloidal properties need to be considered carefully. The experiments here show that IONs with relatively large cores, 16 and 22 nm, can generate high *r*_1_ values. The *r*_1_ values depended on the aggregation state of particle systems. Particles coated with small PEG ligands showed the largest degree of aggregation and lowest *r*_1_ values. Well-separated ION cores with low aggregation had the highest *r*_1_. NMRD measurements showed that the 16 and 22 nm particles had qualitatively different spectra below 2 MHz. The 16 nm IONs showed a peak in *r*_1_ around 1 MHz, whereas the *r*_1_ of the 22 nm IONs increased as the frequency was lowered. These results show that Néel relaxation plays an important role in the low field *T*_1_ relaxation. The Néel relaxation of IONs is related to both the size and magnetic anisotropy of the particles, so these properties should be considered together when designing future contrast agents. To conclude, IONs with dimeters ranging from 16 to 22 nm exhibit high longitudinal relaxivity at 64 mT, making them promising materials for LF-MRI T_1_ contrast agents. Here, 16 nm particles exhibited an *r*_1_ that was an order-of-magnitude larger than a clinical Gd-based agent. However, as the diameter of IONs increases, material parameters such as coating and core size can impact the relaxivity through aggregation and reduced Néel relaxation rates.

## Supplementary Material



## Notes

Synthesis of nitroDOPA (Section S1), synthesis of nitroDOPA-terminated PEG (Section S2), particle size distributions from TEM (Section S3), X-ray diffraction of IONs (Section S4), fitting for determination of relaxivity (Section S5), calculation of PEG length (Section S6), calculation of relaxivity as a function of Larmor frequency (Section S7), estimation of Néel relaxation times (Section S8) (PDF)
