# Supplementary material for: Engineering the Colloidal Properties of Iron Oxide Nanoparticles for High T 1 MRI Contrast at 64 mT
Source: ACS Appl Nano Mater. 2025 Sep 12;8(38):18424–33. doi: 10.1021/acsanm.5c03154 (PMC12481475; doi:10.1021/acsanm.5c03154)
Supplement: Supplementary file 1 [file an5c03154_si_001.pdf]

Supporting Information for "Engineering the Colloidal Properties of Iron Oxide Nanoparticles for High T_1 MRI Contrast at 64 mT"

Samuel D. Oberdick[1,2]*, Gabriella G. Erich[2], Arabella R. Stockdale[3], Kaitlyn M. Betz[2], Kalina V. Jordanova[2,4], Andrew G. Korovich[5], O. Thompson Mefford[3], Giacomo Parigi[6], Megan E. Poorman[7], Gary Zabow[2], Kathryn E. Keenan[2]

Affiliations

1. Department of Physics, University of Colorado, Boulder, CO 80309 USA
2. National Institute of Standards and Technology, Boulder, CO 80305 USA
3. Department of Materials Science and Engineering, Clemson University, Clemson, SC 29634 USA
4. Radiological Sciences Laboratory, Stanford University, Stanford, CA 94305 USA
5. National Institute of Standards and Technology, Gaithersburg, MD 20899 USA
6. Magnetic Resonance Center (CERM), University of Florence, via Luigi Sacconi 6, Sesto Fiorentino, 50019 Italy; Department of Chemistry "Ugo Schiff", University of Florence, via della Lastruccia 3, Sesto Fiorentino, 50019 Italy; and Consorzio Interuniversitario Risonanze Magnetiche Metallo Proteine (CIRMMP), via Luigi Sacconi 6, Sesto Fiorentino, 50019 Italy
7. Hyperfine, Inc. Guilford, CT 06437 USA

*samuel.oberdick@nist.gov

Contents

Section S1. Synthesis of nitroDOPA
Section S2. Synthesis of nitroDOPA-Terminated PEG
Section S3. Particle Size Distributions from TEM
Section S4. X-Ray Diffraction of IONs
Section S5. Fitting for Determination of Relaxivity
Section S6. Calculation of PEG Length
Section S7. Calculation of Relaxivity as a Function of Larmor Frequency
Section S8. Estimation of Néel Relaxation Times

Section S1. Synthesis of nitroDOPA

To create a secure anchor for the polymer to attach to the magnetite surface, 3,4 dihydroxyphenylanaline (DL-DOPA) was nitrated using a method described by Yang et al.[1] Nitrating the catecholamine was performed using an 1.5 mol/L sulfuric acid titration (0.8 mL, 17.8 mol/L acid and 9 mL of deionized water) with DL-DOPA (1.97 g, 9.99 mmol) and sodium nitrite (1.52 g, 17.88 mmol) dissolved in 100 mL of deionized (DI) water. This was performed dropwise in an ice bath at (4 °C monitored via a thermal probe) where the analyte was allowed to equilibrate to prior to the titration. Acid was added at a rate of 0.2 mL/min via an addition funnel while stirring with a magnetic stir bar until a dark yellow precipitate was formed, indicating the presence of nitro- 3,4 dihydroxyphenylanaline (nitroDOPA). The reaction mixture was vacuum filtered and washed in excess with cold methanol before sealing in a desiccator to dry for 4 days. The product was verified by nuclear magnetic resonance spectroscopy (NMR) in deuterated dimethyl sulfoxide (DMSO-d_6) by peaks at 3.02 ppm and 3.25 ppm (doublet of doublets, (ring-CH_2-C)), a triplet at 3.54 ppm ($CHNH_2$), and singlets at 6.81 ppm (CH, ring, nitroDOPA) and 7.42 ppm (CH, ring, nitroDOPA).

Section S2. Synthesis of nitroDOPA-Terminated PEG

In this study, a series of four PEG polymers were modified of weights M_n = 2000 Da, 5000 Da, 10000 Da, and 20000 Da (M_n refers to the number average molecular weight). First, monomethyl ether PEG (1 mmol) with a hydroxy-terminated group was reacted with succinic anhydride (0.225 g, 2.25 mmol) and 4-dimethyl aminopyridine (DMAP) (0.0916 g, 0.75 mmol) in anhydrous tetrohydrofuran (THF) (35 mL) at 40 °C for 12 hours to achieve a monofunctional carboxylic acid-terminated PEG (~70 % yield). The resulting polymer was purified by centrifuging in cold diethyl ether as the antisolvent and redispersing in chloroform three times until the polymer was left to dry in a vacuum oven for 48 hours. The carboxylic acid-terminated PEG (0.6 mmol) was then reacted with N,N'-dicyclohexylcarbodiimide (DCC) (0.149 g, 0.72 mmol) and N-hydroxysuccinimide (NHS) (0.086 g, 0.75 mmol) in anhydrous THF (35 mL) at room temperature for 12 hours yielding an NHS-terminated PEG (90.4% yield). Vacuum filtration was used to remove unreacted impurities, and the resulting solution was purified and dried using the same techniques described above. In the last step, PEG-NHS (0.1 mmol) was reacted with nitroDOPA (0.036 g, 0.15 mmol) in anhydrous dimethylformamide (DMF) (30 mL) to form nitroDOPA-PEG.

This polymer was vacuum filtered, purified with diethyl ether and chloroform under centrifugation and left to dry under vacuum for 48 hours (58% yield). This procedure was used for the 2000 Da, 5000 Da and 20000 Da polymers whereas the 10000 Da polymer was commercially available as an NHS-terminated PEG, eliminating the first two steps of the procedure. Figure S1 shows the synthetic route to PEG-nitroDOPA. NMR spectroscopy (Figure S2) confirmed the modification of nitroDOPA-PEG in deuterated chloroform by peaks at 6.6 ppm, 6.75 ppm (singlets, CH, ring, DOPA), 2.7 ppm, 2.5 ppm (doublet of doublets, ring - CH_2-C, DOPA), 2.47 ppm (coupled triplet, O=C-CH_2-CH_2-C=O, succinic anhydride addition), and 3.65 ppm (O-CH_2-CH_2-O, PEG).

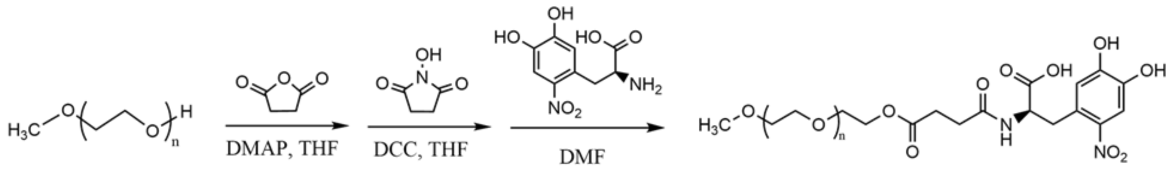

Figure S1. Synthetic route to PEG-nitroDOPA.

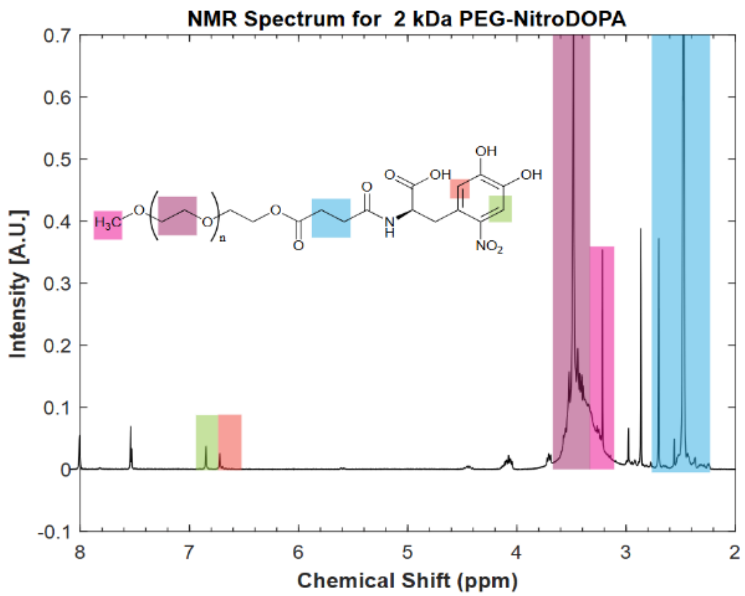

Figure S2. Nuclear magnetic resonance (NMR) spectrum for 2 kDa PEG-NitroDOPA.

Section S3. Particle Size Distributions from TEM

Particle size distributions for the iron oxide nanoparticle (ION) cores were obtained using transmission electron microscopy (TEM) and image analysis. The mean core size and standard deviation were determined from distributions.

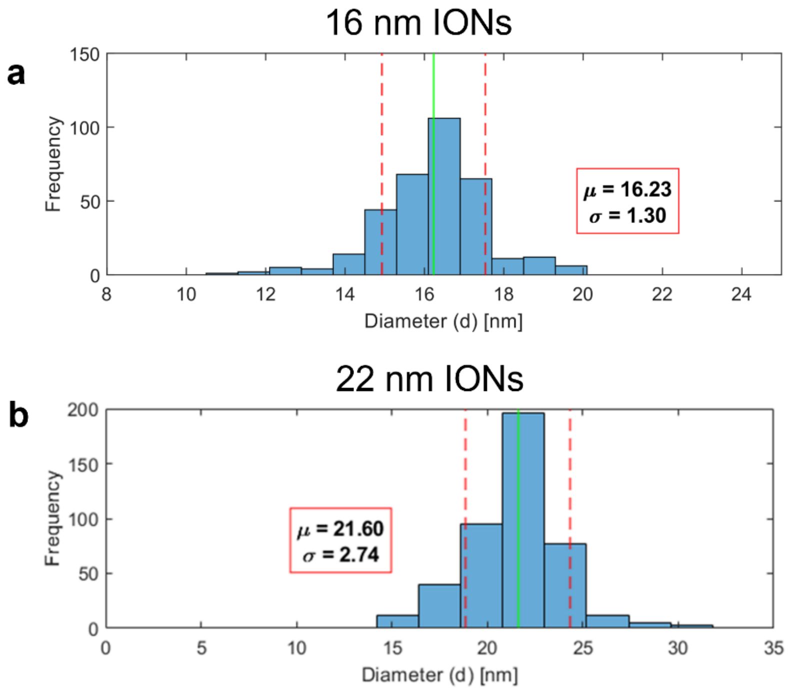

Figure S3. Particle core size distributions for (a) the 16 nm IONs and (b) the 22 nm IONs.

Section S4. X-Ray Diffraction of IONs

X-ray diffraction (XRD) was performed using a Rigaku SmartLab powder X-ray diffractometer. The nanoparticle sample, which was suspended in hexane, was added dropwise to a circular glass pane, and allowed to dry until a level, evenly filled surface was made. The sample was scanned at from 20 degrees to 80 degrees with a speed of 3 degrees/minute.

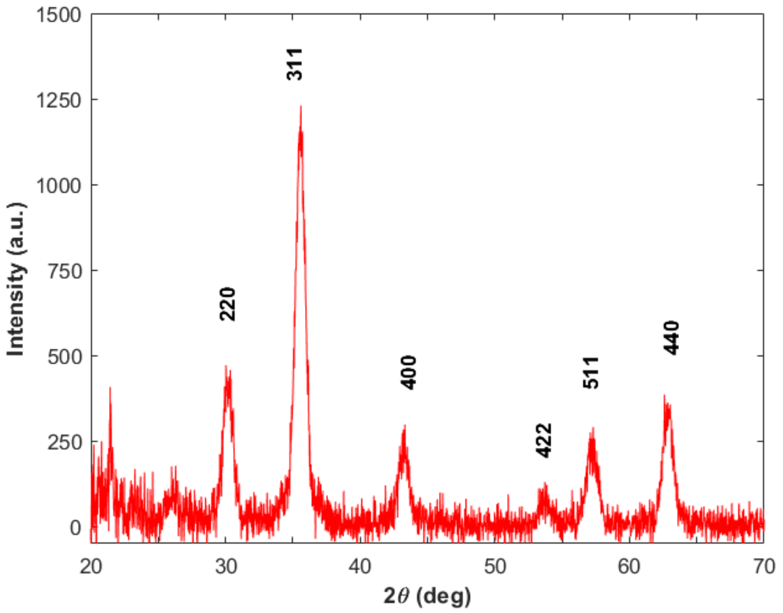

Figure S4. XRD of 16 nm IONs labelled with magnetite peaks.

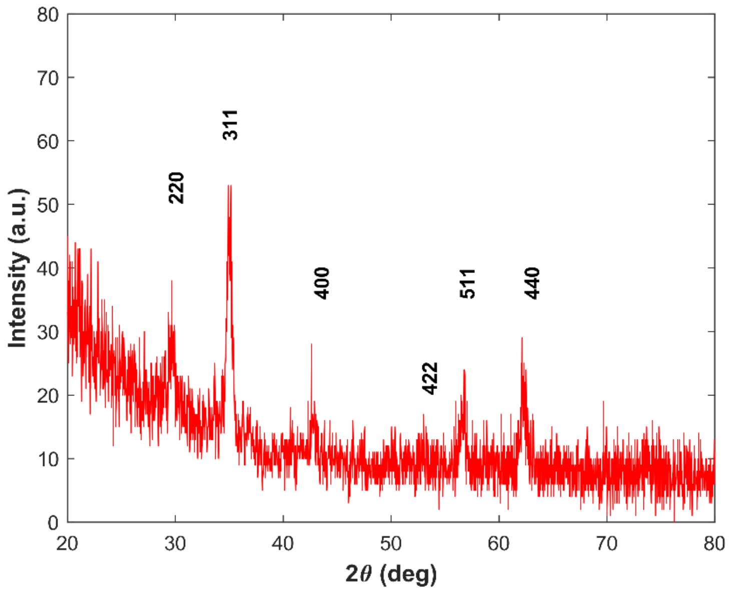

Figure S5. XRD of 22 nm IONs labelled with magnetite peaks.

Section S5. Fitting for Determination of Relaxivity

The longitudinal and transverse relaxivities, r_1 and r_2, were determined by first plotting the proton relaxation rates ($1/T_1$ and $1/T_2$) as a function of sample concentration and then fitting the data to a line. The slope of the line was reported as the relaxivity. The data and fits are displayed in Figure S6 and Figure S7. The data points represent the relaxation rate calculated for each sample at various slices of the MRI data. Two slices from the top and bottom of the sample container were excluded from the fit.

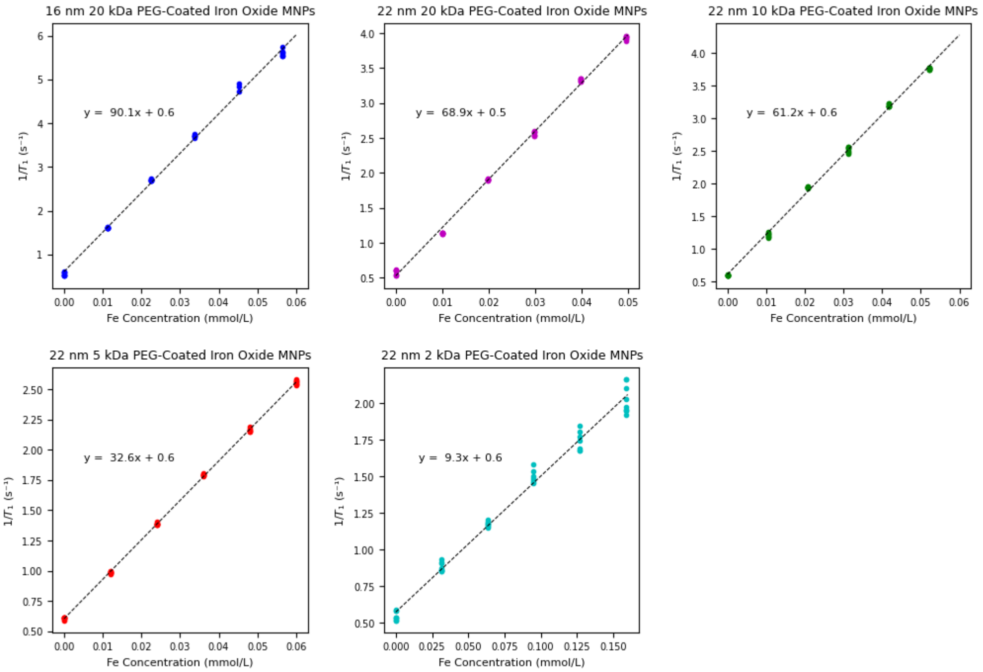

Figure S6. Plots of the longitudinal relaxation rate, $1/T_1$, as a function of iron concentration in the sample. The data was fit to a line and the slope was reported as the longitudinal relaxivity.

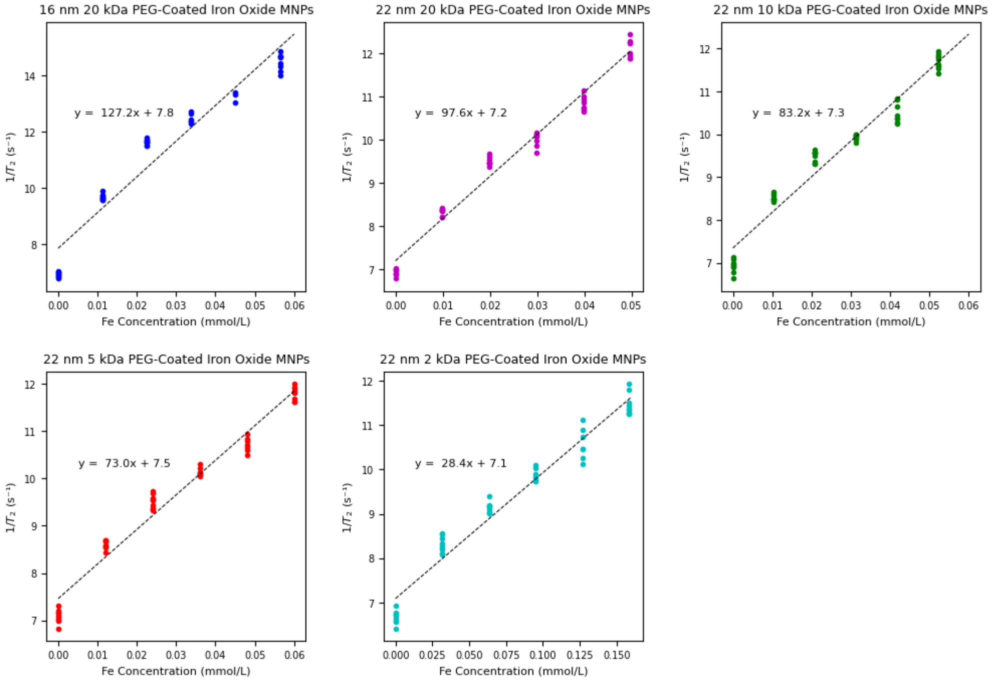

Figure S7. Plots of the transverse relaxation rate, $1/T_2$, as a function of iron concentration in the sample. The data was fit to a line and the slope was reported as the transverse relaxivity.

Section S6. Calculation of PEG Chain Length

In Figure 6b (main manuscript), the theoretical size of particles was calculated using the contour length, which is the product of the length of segments times the number of segments. The calculations in Table S1 are based on a poly(ethylene glycol) (PEG) segment length of 0.28 nm and PEG molecular weight of $44.05n + 18.02$ Da, where n is the number of PEG segments[2].

Table S1. Parameters used to calculate the overall dimeter of the iron oxide nanoparticle (ION) core and fully extended PEG coating.

PEG Mw (Da)	Number of PEG Segments, *n*	Total PEG Length (nm)	Total Diameter (nm) (PEG + ION Core + PEG)
20000	454	127	276
10000	227	63	149
5000	113	32	85
2000	45	13	47

Section S7. Calculation of Relaxivity as a Function of Larmor Frequency

The model from A. Roch et al.[3] was used to calculate the longitudinal relaxivity, r_1, for an experimentally relevant range of Larmor frequencies. The calculation was used to generate the theory curves in Figures 7a and 7b in the main paper. The parameters used to generate the theoretical curve were informed by the structural and magnetic properties of the particles. Tables S2 and S3 contain the values used to generate the curve for the 16 nm and 22 nm iron oxide nanoparticles (IONs), respectively. The spin of the nanoparticles, S, was calculated using experimentally determined values for the magnetization of the IONs.

Table S2. Parameters used to calculate r_1 vs. frequency for 16 nm IONs.

Parameter	Value
Temperature, T	293 K
Diffusion coefficient, D	2.2×10^{-9} m^2/s
Distance of closest approach, d	1.25×10^{-8} m
Néel relaxation time, τ_N	5×10^{-8} s
Particle Concentration, C	1.1×10^{-5} mmol/L
Spin, S	40000

Table S3. Parameters used to calculate r_1 vs. frequency for 22 nm IONs.

Parameter	Value
Temperature, T	293 K
Diffusion coefficient, D	2.2×10^{-9} m^2/s
Distance of closest approach, d	2×10^{-8} m
Néel relaxation time, τ_N	3×10^{-6} s
Particle Concentration, C	4.4×10^{-6} mmol/L
Spin, S	82000

Section S8. Estimation of Néel Relaxation Times

The Néel relaxation times, τ_N, were estimated using the Néel-Arrhenius equation:

$$\tau_N = \tau_0 e^{KV_{mag}/k_B T} \quad \text{Equation S1}$$

In Equation S1, τ_0 is the attempt time (taken to be 10^{-9} s), K is the effective anisotropy constant of the particles, V_{mag} is a re-scaled magnetic volume of the particles, k_B is Boltzmann's constant, and T is the temperature. The magnetic volume of the particles was found by re-scaling the physical

volume, V_{phys} (from TEM), by the ratio of measured magnetization, M_{meas}, to ideal magnetite magnetization, M_{Fe3O4}:

$$V_{mag} = (M_{meas}/M_{Fe3O4})V_{phys} \qquad \text{Equation S2}$$

The volume magnetization (in A/m) of the particles was calculated from the measured mass magnetization (Am^2/kg) by assuming a magnetite (Fe_3O_4) stoichiometry. Since the measured magnetization was lower than ideal Fe_3O_4 for both particles (3.5×10^5 A/m for the 16 nm IONs, 2.7×10^5 A/m for the 22 nm IONs, and 4.8×10^5 A/m for bulk magnetite[4]), the magnetic volume was smaller than the physical volume. The re-scaled magnetic volume was used to calculate the Néel time in Equation S1 to try and capture physical effects such as defects and atomic disorder that can lead to reduced magnetization and a non-ideal Fe_3O_4 crystal lattice. Table S4 shows the parameters used to estimate the Néel relaxation time for each particle:

Table S4. Parameters used to estimate the Néel relaxation time for each iron oxide nanoparticle (ION) sample.

Physical Diameter, d (nm)	Physical Volume, V_{phys} (m^3)	Magnetic Volume, V_{mag} (m^3)	Anisotropy Constant, K (J/m^3)	Attempt Time, τ_0 (s)	Temperature, T (K)	Néel Time, τ_N (s)
16	2.1×10^{-24}	1.6×10^{-24}	1×10^4	1×10^{-9}	293	5×10^{-8}
22	5.6×10^{-24}	3.1×10^{-24}	1×10^4	1×10^{-9}	293	3×10^{-6}

The same anisotropy constant, $K = 1 \times 10^4$ J/m^3, was used for both sizes. The value was chosen based on previous experimental measurements of anisotropy constants for iron oxide nanoparticle systems, which reported K values in the range of 1×10^4 J/m^3 to 2×10^4 J/m^3 [5–7].

References

(1) Yang, X.; Hong, H.; Grailer, J. J.; Rowland, I. J.; Javadi, A.; Hurley, S. A.; Xiao, Y.; Yang, Y.; Zhang, Y.; Nickles, R. J.; Cai, W.; Steeber, D. A.; Gong, S. cRGD-Functionalized, DOX-Conjugated, and ^{64}Cu-Labeled Superparamagnetic Iron Oxide Nanoparticles for Targeted Anticancer Drug Delivery and PET/MR Imaging. *Biomaterials* **2011**, *32* (17), 4151–4160. https://doi.org/10.1016/j.biomaterials.2011.02.006.

(2) Oesterhelt, F.; Rief, M.; Gaub, H. E. Single Molecule Force Spectroscopy by AFM Indicates Helical Structure of Poly(Ethylene-Glycol) in Water. *New J. Phys.* **1999**, *1* (1), 6. https://doi.org/10.1088/1367-2630/1/1/006.

(3) Roch, A.; Muller, R. N.; Gillis, P. Theory of Proton Relaxation Induced by Superparamagnetic Particles. *The Journal of Chemical Physics* **1999**, *110* (11), 5403–5411. https://doi.org/10.1063/1.478435.

(4) Cullity, B. D.; Graham, C. D. *Introduction to Magnetic Materials*; John Wiley & Sons, 2011.

(5) Piotrowski, S. K.; Matty, M. F.; Majetich, S. A. Magnetic Fluctuations in Individual Superparamagnetic Particles. *IEEE Transactions on Magnetics* **2014**, *50* (11), 1–4. https://doi.org/10.1109/TMAG.2014.2321327.

(6) Orozco-Henao, J. M.; Muraca, D.; Sánchez, F. H.; Mendoza Zélis, P. Determination of the Effective Anisotropy of Magnetite/Maghemite Nanoparticles from Mössbauer Effect Spectra. *J. Phys. D: Appl. Phys.* **2022**, *55* (33), 335302. https://doi.org/10.1088/1361-6463/ac708e.

(7) Mamiya, H.; Fukumoto, H.; Cuya Huaman, J. L.; Suzuki, K.; Miyamura, H.; Balachandran, J. Estimation of Magnetic Anisotropy of Individual Magnetite Nanoparticles for Magnetic Hyperthermia. *ACS Nano* **2020**, *14* (7), 8421–8432. https://doi.org/10.1021/acsnano.0c02521.

Disclaimer

Any mention of commercial products is intended solely for experimental detail; it does not imply recommendation or endorsement by NIST.
